# Harnessing NK Cells to Control Metastasis

**DOI:** 10.3390/vaccines10122018

**Published:** 2022-11-25

**Authors:** Xuewen Deng, Hiroshi Terunuma

**Affiliations:** 1 Biotherapy Institute of Japan Inc., 2-4-8 Edagawa, Koto-ku, Tokyo 135-0051, Japan; 2 N2 Clinic Yotsuya, 5F 2-6 Samon-cho, Shinjuku-ku, Tokyo 160-0017, Japan

**Keywords:** tumor, metastasis, circulating tumor cells (CTCs), NK cells, immunotherapy

## Abstract

In recent years, tumor immunotherapy has produced remarkable results in tumor treatment. Nevertheless, its effects are severely limited in patients with low or absent pre-existing T cell immunity. Accordingly, metastasis remains the major cause of tumor-associated death. On the other hand, natural killer (NK) cells have the unique ability to recognize and rapidly act against tumor cells and surveil tumor cell dissemination. The role of NK cells in metastasis prevention is undisputable as an increase in the number of these cells mostly leads to a favorable prognosis. Hence, it is reasonable to consider that successful metastasis involves evasion of NK-cell-mediated immunosurveillance. Therefore, harnessing NK cells to control metastasis is promising. Circulating tumor cells (CTCs) are the seeds for distant metastasis, and the number of CTCs detected in the blood of patients with tumor is associated with a worse prognosis, whereas NK cells can eliminate highly motile CTCs especially in the blood. Here, we review the role of NK cells during metastasis, particularly the specific interactions of NK cells with CTCs, which may provide essential clues on how to harness the power of NK cells against tumor metastasis. As a result, a new way to prevent or treat metastatic tumor may be developed.

## 1. Introduction

Although tumor treatment has improved, most metastatic tumors remain incurable [1]. Metastasis is the major cause of tumor-associated death [2]. Hence, concerted efforts have been made to develop therapies that can control metastasis in patients with tumor [3]. Tumors are complex ecosystems composed of neoplastic cells, extracellular matrix (ECM), and “accessory” non-neoplastic cells, which include resident mesenchymal cells, endothelial cells, and immune cells. Accessory cells crosstalk with tumor cells, which fuels and shapes tumor development [4]. Immune cells are essential players in tumor, impacting tumor fate in different stages and therapeutic interventions [4,5]. Over the past decades, immunotherapy has revolutionized tumor treatment [6]. The emergence of immune checkpoint inhibitors (ICIs) targeting programmed cell death protein 1 (PD-1), cytotoxic T lymphocyte-associated protein (CTLA-4), and programmed cell death ligand 1 (PD-L1) has rendered many aggressive tumors treatable and even curable [6]. However, ICIs are successfully used in only a fraction of patients. In particular, their effects are severely limited in patients with low or absent pre-existing T cell immunity [7]. Thus, the initiation of a de novo tumor-specific immune response is required, a process that is dependent on the actions of innate immune cells, among which natural killer (NK) cells play essential roles in tumor immunosurveillance and antitumor immunity owing to their unique ability to identify and kill tumor cells [8]. Indeed, a higher infiltration of NK cells was found to be associated with the better response to anti-PD-1 therapy [9]. NK cells might play an important role in the tumor subsets that exhibit loss of neoantigen presentation due to the downregulation of major histocompatibility complex class I (MHC-I) molecules [10]. Currently, tumor immunotherapy based on NK cells is a very hot topic in oncology and generates considerable interest from the scientific community and pharmaceutical industry. Consequently, there are many studies in which the therapeutic value of NK cells is being evaluated [11,12]. However, the efficacy of NK cell therapy against solid tumor is hampered by inadequate tumor infiltration and immunosuppressive tumor microenvironment. Since NK cells play a more important role in the elimination of metastatic tumor than primary solid tumors [13], we will focus on the interactions of NK cells with metastatic tumor cells, the prominent role of NK cells in the control of metastasis, and the evasion of metastatic tumor cells from NK-cell-mediated immunosurveillance. Understanding tumor cell resistance to NK cells, particularly the specific interactions of circulating tumor cells (CTCs) with NK cells may provide essential clues on how to harness NK cell power to maximize their antitumor potential and may pave the way to the development of novel therapeutic strategies for metastatic tumor. 

## 2. Tumor Metastasis

**Figure 1:**
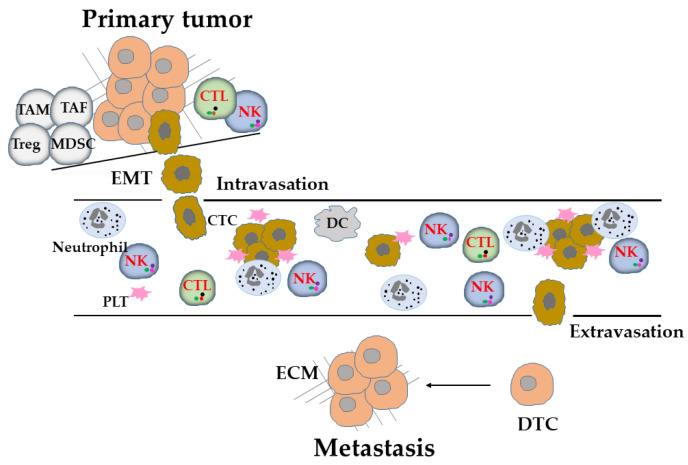
Metastatic cascade and immunosurveillance. Primary tumor cells undergo epithelial-to-mesenchymal transition (EMT) within tumor-cell-extrinsic factors in nutritional, stromal, and immunological microenvironments endowed with distinct metastatic potential. The EMT trans-differentiated cells detach from the primary tumor and interact with and remodel the extracellular matrix (ECM) for intravasation, leaving the protection of the immunosuppressive tumor microenvironment. They reach the circulation, which make them more vulnerable to attacks by immune effector cells, particularly, NK cells, which can directly and indirectly interact with circulating tumor cells (CTCs) to control metastasis. However, they survive as polyclonal clusters of CTCs and cooperate with platelets and neutrophils, which shield them and alter the function of NK cells as a mode of escape from immunosurveillance until they encounter conditions that are permissive for extravasation and lodge at secondary sites. They can persist in a relatively silent state as disseminated tumor cells (DTCs) or micrometastatic tumor cells for a highly variable period of time, until they resume proliferation to establish a clinically detectable metastatic disease. Abbreviations: TAF, tumor-associated fibroblast; CTC, circulating tumor cell; CTL, cytotoxic T lymphocyte; DC, dendritic cell; DTC, disseminated tumor cell; ECM, extracellular matrix; EMT, epithelial-mesenchymal transition; MDSC, myeloid-derived suppressor cell; NK, natural killer cell; PLT, platelet; TAM, tumor-associated macrophage; Treg, regulatory T cell.

Metastasis, which is the gravest stage of tumor, occurs when tumor cells acquire invasive features [14] and the ability to evade immunosurveillance [15]. For the successful outgrowth of tumor cells to distant metastatic sites, several critical steps and obstacles need to be overcome. This multistep process, known as the metastatic cascade, involves the detachment of tumor cells from their neighbors and local invasion of surrounding tissues to enter the circulatory system as CTCs in the form of either single (monoclonal) or multiple (polyclonal) tumor cells, until they lodge at secondary sites and enter into a pre-existing or neo-formed vasculature and remain there as disseminated tumor cells (DTCs) or micrometastatic sites (Figure 1). DTCs remain dormant until they resume proliferation and establish detectable metastatic lesions, giving rise to overt macrometastasis and organ colonization [16]. During this process, mesenchymal-like phenotype tumor cells have highly mobile and invasive properties, which occur during the so-called epithelial-to-mesenchymal transition (EMT) [17]. Activation of the EMT program impinges on the immunomodulatory properties and immunogenicity of tumor cells [18,19,20]. CTCs must perform well in multiple events for successful metastasis, especially for successful in survival in the circulation [21] (Figure 1). Most of the CTCs are shed from the primary tumor site, which has become an immunosuppressive microenvironment that protects them from immune attacks, and die during their transport in blood vessels because of hemodynamic shear force, oxidative stress, and susceptibility to immune effector cells [22,23] (Figure 1). Ultimately, only a very small fraction of CTCs survive and become seeds for metastasis. Among tumor-extrinsic factors for metastasis, antitumor immunity, which is a major hindrance to metastatic colonization of CTCs and DTCs [22,23] (Figure 1). Polyclonal CTCs, which have higher metastatic potential [24,25] because they are associated with decreased expression levels of NK-cell-activating ligands that exhibit higher resistance to killing by NK cells [26]. Conventionally, metastasis has been considered to occur in later stages of tumor progression. However, accumulating evidence has also shown metastatic tumor cell dissemination during early tumor formation [27]. The metastatic cascade and immunosurveillance involving NK cells in metastasis are illustrated in Figure 1.

## 3. NK Cells Are Highly Involved in for the Immunosurveillance of Metastasis

NK cells are effector cells that constitute a key part of the innate immune system and represent up to 5–20% of circulating lymphocyte [28,29]. NK cells show an absence of CD3 molecule but highly express CD56 or CD16, are predominantly defined as CD3^−^CD56^+^. There are two major types of NK cell: CD56^dim^CD16^bright/+^ (CD56^dim^) and CD56^bright^CD16^dim/−^ (CD56^bright^) NK cells. The CD56^dim^ NK cells are the major subset in peripheral blood, have high cytotoxicity, whereas CD56^bright^ NK cells are predominantly in secondary lymphoid organs as cytokine producing NK cells [28,29]. NK cells have the unique ability to differentiate between normal and transformed cells, and they can recognize and rapidly act against malignant cells without prior sensitization [28,29]. They possess various activating and inhibitory receptors, and the net functional outcome is a complex integration of signals among these activating and inhibitory receptors [28,29]. These inhibitory receptors, such as the inhibitory isoforms of killer-cell immunoglobulin receptors (KIRs), and CD94/NKG2A heterodimers recognize various forms of MHC-I molecules. Thus, the decrease in the expression levels or the absence of MHC-I molecules on tumor cells reduces the strength of inhibitory signals delivered to NK cells, thus promoting NK cell activation. NK cell activation also results from the engagement of activating receptors, such as the activating isoforms of KIRs, the signaling lymphocyte-activating molecule-related receptors NKG2D, DNAX accessory molecule-1 (DNAM-1), and the natural cytotoxicity receptors NKp30, NKp44, and NKp46, which recognize stress-inducible ligands on tumor cells that are scarcely expressed in healthy cells. Therefore, NK cells have a well-documented antitumor effect, including antibody-dependent cellular cytotoxicity (ADCC) through specific IgG antibodies to target antigens [28,29,30,31]. Indeed, an epidemiological study has shown that a reduced NK cell function was associated with an increased tumor incidence in humans [32].

When NK cells recognize aberrant cells, such as tumor cells, they are activated and transport specific lytic granules such as perforins and granzymes toward immunological synapses to induce apoptosis of target cells [33]. Perforins are cytolytic proteins that are inserted into the plasma membrane of a target cell and induce osmotic lysis in a Ca^2+^-dependent manner [34]. Granzymes are serine proteases that activate caspase signaling, leading to the apoptosis of the target cell [35]. Perforins are critical in controlling tumor metastasis [36,37]. NK cells form multiple contacts with target cells and can sequentially kill several tumor cells in a time-dependent manner [38]. Interestingly, upon a single encounter, an NK cell releases only one tenth of its cytotoxic lytic granules, but it has been determined that even a single granule is sufficient to induce tumor cell death [34]. NK cells have been shown to shift from inducing fast granzyme B-mediated cell death to slow death mediated by receptor ligands (Fas ligand and tumor necrosis factor (TNF)-related apoptosis-inducing ligand (TRAIL)) in later stages and can serially kill up to more than 30 tumor cells [39].

Although the role of NK cells in the control of primary tumors remains controversial, their robust antimetastatic effect in vivo has been demonstrated in various experimental models [40,41,42,43,44]. Tumor stem cells (TSCs) [45] and EMT trans-differentiated cells [17] are considered responsible for tumor metastasis. Meanwhile, NK cells can target TSCs and EMT cells [30,46,47,48]. Human and mouse breast TSCs induce NK cell activation and expansion in vivo, which correlate with the inhibition of TSCs metastatic spread [46]. EMT induces E-cadherin- and cell adhesion molecule 1 (CADM1)-mediated NK cell susceptibility. Thus, a higher CADM1 expression level correlates with improved survival and reduced metastasis in patients with lung and breast adenocarcinomas [48]. In human tumors, there is accumulating evidence of correlations among the number of infiltrating NK cells, metastasis, and the prognosis of various tumors, such as esophageal tumor (ET) [49], gastric carcinomas (GCs) [50], gastrointestinal sarcoma tumors [51,52], renal cell carcinomas (RCCs) [53], colorectal carcinomas (CRCs) [54], and prostate tumor (PT-1) [55]. In addition, the presence of highly effective NK cells indicates a good prognosis in metastatic PT-1 [56]. Overall, an increased number of NK cells is highly beneficial for the survival of tumor patients [8]. Importantly, however, CTCs are considered to be indicators of an increased risk of metastasis and poorer outcomes in tumor patients. In particular, NK cells are present in blood and lymph nodes and may participate in the immunosurveillance of CTCs [22,44]. In an experimental model, NK cells destroy CTCs before the extravasation and thus suppressing tumor metastasis [42,44,57]. Thus, a short period of an elevated number of NK cells is correlated with the reduction in the number of CTCs [58,59]. Indeed, NK cells were found to eliminate disseminated tumor cells from the lungs within 24 h of arrival, but not thereafter [44]. Moreover, the cytotoxicity of NK cells from CTC-positive patients is lower than that from CTC-negative patients with metastatic breast tumor (BT), CRC, and PT-1, showing a close correlation between peripheral blood CTC number and NK cell antitumor activity [60,61]. Hence, a decreased circulating NK cell activity is associated with increased risk of metastasis in patients with pharyngeal carcinoma [62]. In addition, a decreased NK cell activity is a parameter for predicting distant metastasis following curative surgery for CRC [63,64,65]. Moreover, surgical stress induces NK cell dysfunction in animal models [66,67,68], and human tumors [69,70,71,72,73], leading to metastasis [66,67,68]. Thus, enhancing NK cell function by inhibiting the induced NK cell dysfunction can prevent postoperative metastasis [66,67,68,74]. Taken together, these results indicate that NK cells eliminate CTCs and play a prominent role in the control of metastasis [13].

## 4. Evasion of NK-Cell-Mediated Immunosurveillance in Metastasis 

**Figure 2:**
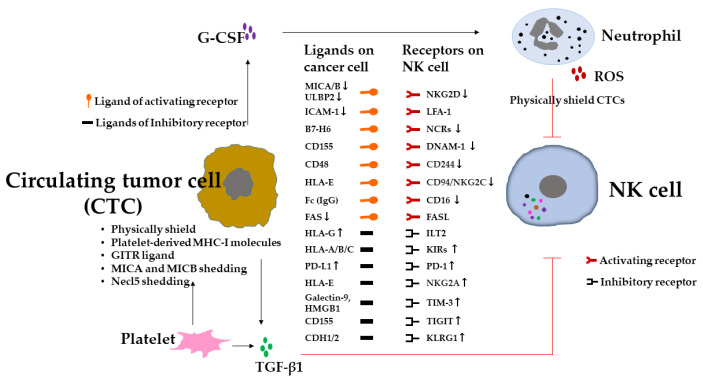
Circulating tumor cells (CTCs) evade surveilling NK cells during metastasis. For successfully metastasis, tumor cells have to evade from NK-cell-mediated immunosurveillance. Tumor cells have several strategies involving tumor cell intrinsic features and tumor-driven extrinsic microenviroment factors to subvert their recognition and elimination by NK cells. Intrinsic features endow tumor cells with distinct metastatic potential owing to epigenetic and genetic alterations that include the downregulation of NK cell activating receptor ligands and/or the upregulation of NK cell inhibitory receptor ligands. In contrast, the tumor-cell-driven extrinsic microenvironment suppresses NK cell immunosurveillance through modulation of activating receptors and/or inhibitory receptors on NK cells. Platelets and neutrophils may favor the escape of CTCs from NK cell immunosurveillance, thereby supporting CTCs to establish metastasis. (Arrows indicate the change in expression levels of surface receptors).

The immune system plays a major role in every step of tumor progression [4,5]. The escape of tumor cells from immunosurveillance is a prerequisite for metastasis [15,23]. Since NK cells are highly involved in for immunosurveillance in metastasis [13], it is therefore plausible that for successful metastasis, tumor cells have to evade NK-cell-mediated immunosurveillance. Indeed, several mechanisms of NK-cell-mediated tumor cell escape have been described [23,26,75]. These mechanisms involve tumor cell intrinsic features and tumor-driven extrinsic microenvironmental factors (Figure 2). Herein, we explore these escape mechanisms of NK cells in metastasis. The intrinsic features endow tumor cells with distinct metastatic potential through epigenetic and genetic alterations, including the downregulation of NK cell activating receptor ligands due to, for example, the reduction in the expression levels of MHC-I polypeptide-related sequence A (MICA)/MICB and UL16-binding protein 2 (ULBP2) caused by the aberrant expression of oncogenic microRNA, miR-20a, and miR-34a/c in tumor cells [76,77]. MICA/MICB and ULBP2 are the ligands of the NK cell activating receptor NKG2D. Along similar lines, tumor cells expressing miR-296-3p inhibit the expression of intercellular adhesion molecule 1 (ICAM-1), which is the ligand of β2 integrin lymphocyte function-associated antigen 1 (LFA-1), providing activating signals to NK cells [58]. In addition, NK cell inhibitory receptor ligands, such as human leukocyte antigen-G (HLA-G) and PD-L1, are up-regulated. HLA-G is the non-classic MHC-I gene and the ligand for the inhibitory receptor of KIRs, which are highly expressed in numerous tumor cells [78]. HLA-G exerts its function through its binding to immunoglobulin-like transcript 2 (ILT2) and KIRs on NK cells to protect tumor cells from NK cell cytotoxicity [79]. Thus, HLA-G expression is associated with tumor metastasis [80]. Moreover, plasma soluble HLA-G (sHLA-G) in extracellular vesicles was associated with the presence of CTCs and disease progression, indicating that CTCs exploit sHLA-G in the blood to evade NK-cell-mediated immunosurveillance [81]. Moreover, NK cell functions are affected by the inhibitory ligand PD-L1 expressed on tumor cells, which provide inhibitory signals to NK cells through the PD-1/PD-L1 axis, and reduce NK cell cytotoxicity [82]. In addition, cell death receptor FAS expression on tumor cells is down-regulated, leading to escape from NK-cell-related apoptosis [83]. 

In contrast, the tumor cell-driven extrinsic microenvironmental factors suppress NK cell immunosurveillance through modulation of the recruitment, cell surface molecules, or the release of immunosuppressive soluble factors such as TGF-β1 [84,85,86] (Figure 2). NK cells in lung, breast, or prostate tumor tissues displaying altered receptor expression with impaired cytotoxicity compared with NK cells in control tissues [84,85,86]. These alterations have also been observed in peripheral NK cells and associated with disease progression [85,86]. The expression levels of activating receptors on NK cells were decreased: for example, such as CD16 in BT, multiple myeloma (MM), and CRC [86,87,88]; NKp46 in pancreatic tumor (PT-2), GC, CRC, acute myeloid leukemia (AML), and cervical tumor (CC-1) [89,90,91]; NKp44 in BT and AML [92,93]; NKp30 in BT, hepatocellular carcinoma (HCC), PT-2, GC, CRC, chronic lymphocytic leukemia, and CC-1 [86,89,90,91,94]; CD94/NKG2C in AML [91]; NKG2D in BT, lung tumor (LT), CRC, colon carcinoma (CC-2), PT-2, GC, and CC-1 [86,89,90,95,96]; CD244 in AML [91]; DNAM-1 in BT, CC-2, and AML [86,91]. On the other hand, the expression levels of inhibitory receptors on NK cells were increased: for example, KIR3DL1 in PT-2, GC, and CRC [84]; KIR2DL2/L3 in melanoma [97]; NKG2A in BT, LT, and CRC [86,98,99]; PD-1 in ET, HCC, CRC, GC, biliary tumor, and LT [100,101]; TIM-3 in LT, melanomas, and GC [102,103,104]. TGF-β1 in the tumor environment is involved in the phenotype alteration and functional impairment of NK cells [85,86]. In addition, TGF-β1 and other immunosuppressive factors produced by tumor cells have been shown to suppress NK cell effector functions [105] (Figure 2) and recruit immunosuppressive cells to tumor tissue, such as regulatory T cells (Tregs) [106], myeloid-derived suppressor cells (MDSCs) [107], CD11b^+^Ly6G^+^ neutrophils [108], and indoleamine 2, 3-dioxygenase 1 (IDO1)-expressing dendritic cells (DCs) [109]. They also contribute to the phenotype alteration and functional impairment of NK cells to prepare for distant microenvironments for metastatic niches (Figure 1). Moreover, neutrophils can cooperate with metastatic tumor cells to enhance both the dissemination of tumor cells out of the primary tumor and their subsequent intravasation into the lung vasculature through the secretion of G-CSF to attract neutrophils, which suppress NK cell activity through ROS signaling and shield intraluminal metastatic tumor cells from being cleared by NK cells (Figure 2), and ultimately enhance metastatic outgrowth [109,110]. Furthermore, tumor cells may reprogram NK cells through DNA methyltransferases (Dnmt1, Dnmt3a, and Dnmt3b), thereby increasing the expression level of inhibitory receptors TIGIT and KLRG1 on NK cells and promoting metastatic outgrowth [111]. 

Platelets are small, anucleated cell fragments that have a characteristic discoid shape and diameters ranging from 1 to 3 μm [112]. The main roles of platelets are the maintenance of the hemostasis of the vascular system and the promotion of wound healing at sites of vascular injury [113]. However, having a high platelet count was identified as a risk factor for adverse outcomes in numerous different tumors [114]. Platelets may affect many components of antitumor immunity [115]. An increased coagulability of platelets facilitates vascular evasion and the establishment of metastasis [114,116], whereas the abrogation of platelet function results in reduced metastasis, depending on the presence of NK cells [117]. Platelets help tumor cells evade NK cell immunosurveillance in the blood stream, supporting CTCs to establish metastasis [114,116] (Figure 1). Platelets promote metastasis by coating CTCs traveling through the blood, thereby physically shielding them from shear force. Moreover, a ‘pseudonormal’ phenotype is conferred to CTCs by the transfer of platelet-derived MHC-I molecules, which causes CTCs to mimic host cells and protects them from recognition by NK cells. Moreover, platelet-induced NK cell inhibitory ligands, such as TNF family members of glucocorticoid-induced TNF receptor-related (GITR) ligands, and NKG2D ligands shedding, particularly MICA and MICB on CTCs, protect CTCs from NK cell immunosurveillance [118,119,120,121] (Figure 2). In addition, platelets may play a prometastatic role by promoting thrombin activation resulting in shedding the DNAM-1 ligand Necl5 from the metastatic tumor cells to evade NK cell surveillance [44]. Moreover, platelets release soluble factors upon activation when adhesion to CTCs. TGF-β1 is one of the platelet-derived soluble factors that inhibit NK cell function partially mediated by the downregulation of the activating receptor NKG2D on NK cells [122] (Figure 2). TGF-β1 initiates and maintains the EMT phenotype alteration of CTCs [123]. Therefore, platelets affect NK cell function on several different levels, and both soluble and membrane-bound factors are involved in the evasion of CTCs from NK cell immunosurveillance in vivo.

Taken together, these results exemplify the obstacles posed by NK cell immunosurveillance in metastasis (Figure 2). The characterization of diverse mechanisms contributing to the dysfunction of NK cells in different tumors may pave the way for the development of novel therapeutic strategies by harnessing NK cells in the control of metastasis. 

## 5. Harnessing NK Cells in Control of Metastasis 

**Figure 3:**
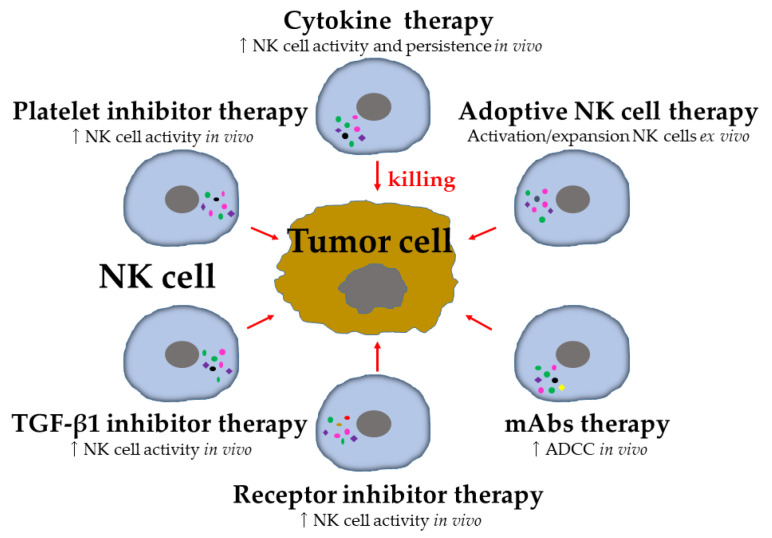
Strategies to harness NK cells in control of metastasis. Each therapeutic strategy is evaluated in isolation or in combination, and even in combination with other strategies including standard treatment. Abbreviations: ADCC, antibody-dependent cellular cytotoxicity; mAbs, monoclonal antibodies. (Arrows indicate enhancement).

A breakthrough in tumor immunotherapy comes from the clinical successes of ICI and chimeric antigen receptor (CAR) T cell therapies, proving that these treatments have great promise for tumor patients [6,124]. NK-cell-based tumor therapy currently constitutes a major area of immunotherapy innovation and has grown exponentially [11,12,125]. To cure tumors, unleashing the full antitumor potential of NK cells is an attractive option (Figure 3) (Table 1), since NK cells play a more important role in the elimination of metastasis [13], which is the major cause of tumor-related death [2]. Most of these strategies are very often reviewed in detail [11,12,13], and the advantage/disadvantages for targeting metastasis are summarized by Lorenzo-Herrero et al. [126].

### 5.1. Cytokine-Based Therapy

Cytokines endow NK cells with enhanced effector functions in antitumor immunity and enhance their persistence in vivo [127] (Figure 3). IL-2 and IL-15 are key cytokines that upregulate the activity of NK cells [128]. IL-2 was the first cytokine employed in tumor treatment and the first reproducible and effective human tumor immunotherapy approved by the Food and Drug Administration (FDA) [129]. An improved survival of patients with metastatic RCC and melanomas was achieved by high-dose IL-2 treatment [130]. However, it caused life-threatening toxicities, including vascular leak syndrome [129,130]. In addition, IL-2 therapy induces the proliferation and activation of Tregs and therefore immunosuppression [131]. Thus, it has been speculated that the limited efficacy of IL-2 therapies in vivo is at least partially attributable to the inhibitory effect of Tregs. For these reasons, there is a need for alternatives to IL-2 and a rationale for the evaluation of variant forms of recombinant IL-2 in order to gain a higher affinity to NK cells and lower affinity to Tregs [132]. Over the past decades, IL-15 has emerged as a promising substitute for IL-2. IL-2 and IL-15 are closely related homeostatic cytokines, and both require CD132 and CD122 heterodimers for signaling [128]. Clinical trials with recombinant IL-15 or IL-15/IL-15Rα complexes are being conducted in metastatic tumors [133] (Table 1). In the first in-human phase I clinical trial of IL-15 in patients with metastatic malignant melanoma and metastatic RCC, hyperproliferation and an increase in the number of circulating NK cells were observed [133]. Even though a preliminary antitumor evaluation showed no objective responses, two patients showed clearance of lung metastasis [133]. Other cytokines that activate NK cells without stimulating Tregs, such as IL-12, IL-18, and IL-21, are now being studied [127]. However, cytokine-based-NK cell-activating strategies in the treatment of metastasis remain to be established.

### 5.2. Adoptive NK Cell Therapy

An alternative approach to the systemic activation of NK cells is to directly introduce activated NK cells to a patient, known as adoptive transfer (Figure 3) (Table 1). As reviewed by Myers et al. [11], a wide variety of sources of therapeutic NK cells are currently being tested clinically, including autologous NK cells, allogenic NK cells, umbilical cord blood (UCB) NK cells, NK cell lines, cytokine-induced memory-like (CIML) NK cells, and CAR NK cells. The adoptive transfer of a patients’ own NK cells (autologous transfer) enables ex vivo stimulation and expansion prior to re-administration as a therapeutic modality. Initial clinical results of adoptive transfer of expanded autologous NK cells in patients with metastatic tumor showed that this treatment strategy is well tolerated, but the clinical response was limited [129,134]. It was considered that the failure of autologous NK cell therapies could be partially attributed to the inability of inhibitory KIRs to recognize self-MHC I on tumor cells. Thus, the adoptive transfer of ‘foreign’ NK cells (allogenic NK cells) for therapy has been examined in patients with various tumors. Allogeneic NK cells derived from healthy donor cells are advantageous because they have higher potential in antitumor activity. A complete remission and disease-free survival have been observed in patients with AML after the adoptive transfer of allogenic KIR-ligand mismatched donor NK cells [135]. The lack of engagement of inhibitory KIR receptors on allogenic NK cells with MHC-I ligands in these patients may be beneficial and contribute to the clinical response [135]. Thus, the transfer of allogeneic NK cells results in a reduction in the CTC number in patients with stage IV non-small cell lung cancer (NSCLC) [136] and recurrent BT [137]. However, the clinical response is limited in metastatic tumor patients in studies of adoptive allogenic NK cell therapy [138,139,140,141,142,143]. To expand the therapeutic use of allogenic NK cells, the use of UCB is considered. NK cells constitute up to 30% of the lymphocytes in UCB, which is a robust source of therapeutic NK cells [144]. The therapeutic efficacy of UCB NK cells is currently being evaluated in clinical trial (Table 1). Clonal NK cell lines, such as NK-92 and KHYG-1, are an alternative source of allogeneic NK cells. The NK-92 cell line has received FDA approval for use in clinical trials and has been extensively tested in clinical trials [145]. However, these cells are aneuploid and therefore genetically unstable, which requires them to be irradiated prior to infusion. NK cells have innate memory, that is, they can remember a prior activation event and consequently respond more robustly when restimulated. NK cell memory has been identified following combined cytokine of IL-12, IL-15, and IL-18 preactivation [146]. The CIML NK cells were shown to have a phenotype distinct from conventional NK cells. They have improved effector functions and induce remission in AML patients [147]. Following the clinical successes achieved with CAR T cell therapies, the use of CAR NK cells represents a promising immunotherapeutic strategy, and CAR NK cells might show greater advantages than CAR T cells, such as the induction of less severe side effects [148]. In this regard, it has recently been shown that CAR NK cells do not cause any serious cytokine storm in patients with lymphoid tumors [149]. In a murine model, CAR NK cell therapy reduced lung metastasis in an RCC by targeting ERbB2/HER2, indicating its potential in the control of disease dissemination [150].

### 5.3. Agonists of Activating and Inhibitory Receptors

NK cell immunosurveillance may be improved in metastasis by targeting NK-cell-function-related activating and inhibitory receptors (Figure 3) (Table 1). Over the past two decades, monoclonal antibodies (mAbs) have been widely used in tumor treatment, and NK cell activity has been increased by employing tumor-specific antibodies that ligate to CD16 receptors on NK cells to promote ADCC [151]. The CD20-targeting mAb rituximab, the epidermal growth factor receptor (EGFR)-targeting mAb cetuximab, and the erb-b2 receptor tyrosine kinase 2 (ERBB2)-targeting mAb trastuzumb are used in the treatment of solid and hematological tumors [152]. The CD38-targeting mAb daratunumb [153] and the CD139-targeting mAb elotuzumab [154] constitute the treatment arsenal against MM. The clinical response is modulated by the polymorphism in the genes encoding CD16 receptors [155], indicating that ADCC plays a crucial role in the therapeutic activity of these mAbs. Children with neuroblastoma are given allogeneic NK cells following administration of anti-GD2 mAb, which recognizes a surface molecule highly expressed in neuroblastoma cells. Administration of NK cells with ADCC enhanced by anti-GD2 mAb produced a partial or complete response in approximately 40% of patients [156,157]. Trastuzumab was successfully introduced in the treatment of HER2^+^ BT and GC. In patients with HER2^+^ BT, even trastuzumab monotherapy has produced a clinical response [158], whereas a clinical study of adoptive infusion of autologous NK cells in addition to trastuzumab in a refractory patient with HER2^+^ BT showed the reversal of the resistance to trastuzumab [159]. Clinical trials on the combinations of autologous NK cells with mAbs are ongoing, such as the combinations of autologous NK cells with trastuzumab in HER2^+^ tumors [160] and with cetuximab in recurrent and/or metastatic nasopharyngeal carcinoma [161] (Table 1). The combination of adoptive NK cells and mAbs targeting CTCs via NK-cell-mediated ADCC is a promising therapeutic strategy for the control of metastasis, as this function appears to be poorly affected by the barrier of the tumor microenvironment. Moreover, CD16 can also be engaged with bispecific or trispecific killer-cell engagers (BiKE or TriKE), which bind the CD16 and tumor antigens simultaneously, to trigger NK cell activation through the CD16 receptor, significantly increasing NK cell cytolytic activity and cytokine production against tumor targets [162].

Blocking inhibitory receptors on NK cells is a suitable strategy to increase antitumor activity [11] (Figure 3) (Table 1). The blockade of the anti-KIR antibody lirilumab enhanced NK cell activity through the blockade of NK cell interaction between inhibitory receptors KIR2DL-1, KIR2DL-2, and KIR2DL-3 with HLA-C group 1 and 2 allotypes [163]. A phase I trial in patients with relapsed/refractory MM using lirilumab as a single agent has shown enhanced ex vivo patient-derived NK cell cytotoxicity against MM (Table 1). However, no objective responses were observed [164]. Therefore, the combination of IPH2101 with lenalidomide is under clinical investigation [165] (Table 1). In addition, the humanized anti-NKG2A antibody monalizumab, which blocks NKG2A–HLA-E interaction, enhanced NK cell activity against various tumor cells; its clinical trials are ongoing [166] (Table 1). Monalizumab monotherapy for recurrent metastatic squamous cell carcinoma of the head and neck was much less efficacious with no objective response, and the disease was stable in only 23% of the patients [167]. On the other hand, checkpoint blockade targeting the PD-1/PD-L1 and CTLA-4 inhibitory axis has produced remarkable results in tumor treatment, and the clinical benefits are considered to mainly be based on the reactivation of exhausted T cells [168]. However, the effect of ICIs on NK cells through PD-1/PD-L1 blockade also mediates antitumor immunity [9]. Thus, targeting PD-1 by blocking the PD-1/PD-L1 signaling axis enhances the NK cell immune response against MM [169]. Clinical studies have been conducted to investigate the effect of pembrolizumab (anti-PD-1) combined with autologous [170] or allogeneic [171] NK cells in patients with NSCLC. These studies showed increased NK cell activity in patients receiving NK cells and improved survival compared with patients receiving pembrolizumab alone [170,171]. Additionally, a fully humanized anti-PD-L1 mAb has been shown to block PD-1/PD-L1 interactions and promote NK-cell-mediated ADCC against tumor cells [172]. Furthermore, TIM-3 blockade has been shown to reverse NK cell impairments and increase NK cell antitumor activity in patients with advanced melanoma and lung adenocarcinoma [102,104]. A plethora of anti-TIGIT mAbs are in advanced clinical development for solid tumors, such as domvanalimb, vibostolimb, tiragolumab, and ociperlimab [173] (Table 1). Further investigation, however, is required to elucidate the actual anti-metastatic potential of blocking inhibitory receptors in humans.

TGF-β1 is one of the major suppressive cytokines produced by tumor cells and platelets. It inhibits NK cell effector functions and helps tumor cells evade NK cell immunosurveillance [84,85,86,123] (Figure 2). A pharmacological inhibitor of the TGF-β1 pathway (Figure 3), galunisertib, has been tested in patients with neuroblastoma [174] and HCC [175], and an increased NK cell activity against tumor cells was observed [174]. 

As platelets can protect tumor cells from NK cell immunosurveillance, targeting the protective interaction of platelets with tumor cells has been suggested to improve the NK cell antitumor activity [114] (Figure 3). The platelet inhibitor ticagrelor specifically inhibits tumor-associated platelets and strongly reduces lung metastasis in a mammary carcinoma mouse model [176]. In addition, silencing tumor-specific tissue factors (TFs) by nanoparticle-mediated delivery of siRNA resulted in reduced platelet adhesion and ultimately the number of lung metastases [177]. Future research will show if targeting the interaction of platelets with tumor cells can improve the efficiency of NK cell immunosurveillance. 

## 6. Conclusions and Future Direction

Tumor immunotherapy by revitalizing immune responses against tumor cells has shifted the paradigm in tumor therapy [6,168]. Nevertheless, the metastatic spread of tumor cells remains the main cause of tumor-related death [2]. NK cells are innate immune cells that can directly and rapidly kill tumor cells without antigen restriction, and they are highly responsible for immunosurveillance in metastasis [13,44]. Hence, tumor cells have to evade NK-cell-mediated immunosurveillance for successful metastasis (Figure 2). Thus, it is promising to harness NK cells for the prevention or treatment of tumor metastasis (Figure 3). As the evading mechanisms involved NK cell immunosurveillance in the metastatic cascade, particularly the specific interactions of CTCs with NK cells in metastasis (Figure 2), an optimal therapeutic window may exist to achieve maximal NK cell antitumor activity. The experimental lung metastasis model showed that NK cells eliminate CTCs from the lung within 24 h of arrival, but not thereafter. Half of NK–tumor cell encounters lead to tumor cell death in the first 4 h after tumor cell arrival [44]. NK cell therapies would be mostly used in the adjuvant setting as adjuvants, such as after surgery [74] and following stem cell transplantation [178] targeting minimal residual disease. Specifically, NK cells may be most effective for targeting CTCs, as their function appears to be poorly affected by the barrier of the tumor microenvironment [179]. Clinical trials are underway to evaluate the efficacy of NK cell immunotherapy by each therapeutic strategy alone or in combination (Figure 3), and further in combination with other strategies, including standard treatments, such as mAbs therapy [160,161] and chemoradiotherapy [180], with encouraging clinical results. Moreover, recent data suggest that molecularly targeted agents [181] and radiotherapy [182] capable of inducing senescence in tumor cells elicit NK-cell-mediated immunosurveillance, facilitating tumor regression, indicating that NK cells are likely to be a key player in future multimodal strategies against tumors. Further studies should be performed in order to fully understand the antitumor and antimetastatic properties of NK cells, particularly when and at which steps of the metastatic cascade NK cells operate, and how many times NK cell immunotherapy is required, which will pave the way to developing novel therapeutic strategies for the prevention or treatment of tumor metastasis.

## Figures and Tables

**Table 1:** Selected clinical trials of NK-cell-based tumor therapy. Data source ClinicalTrials.gov (www.clinicaltrials.gov, accessed on 18 November 2022).

Agent	Approach	Tumor Type	Phase	Trial Identifier
*Cytokine-based therapy*				
N-803 (IL-15 superagonist)	Monotherapy	Advanced-stage melanoma, NSCLC, RCC, HNSCC	I	NCT01946789
NIZ985 (solube IL-15)/IL-15 receptor α heterodimer (hetIL-15)	In combination with spartalizumb (anti-PD-1 antibody)	Solid tumours, lymphoma or melanoma	I/Ib	NCT04261439
*Adoptive NK cell therapy*				
Autologous NK cells	After treatment with bortezomib	Solid and haematological tumor	I	NCT00720785
Allogeneic NK cells	In combination with trastuzumab or cetuximab	Her2^+^ or EGFR^+^ solid tumors	I	NCT03319459
UCB NK cells	Before autologous HSCT	NHL	I/II	NCT03579927
NK-92 cells expressing high-affinity variant of CD16	Combined with N-803 and avelumab	Merkel cell carcinoma that has progressed after ICI	I	NCT03853317
CIML NK cells	Combined with N-803	R/R AML	II	NCT02782546
iC9/CD19-CAR-CD28-zeta-2A-IL-15 NK cells	In combination with lymphodepleting chemotherapy	CD19^+^ R/R B cell lymphoma	I/II	NCT03056339
*Monoclonal antibody therapy*				
Haploidentical NK cells	After treatment with anti-GD2 mAb	Neuroblastoma	I	NCT02650648
Expanded autologous NK cells	After treatment with cetuximab	EGFR^+^ NPC or HNSCC	I/II	NCT02507154
Expanded autologous NK cells	After treatment with trastuzumab	HER2^+^ breast or gastric cancer	I/II	NCT02030561
*Receptor inhibitor therapy*				
Lirilumab (anti-KIR antibody)	In combination with elotuzumab or urelumab	Multiple Myeloma	I	NCT02252263
IPH2101 (anti-KIR antibody)	In combination with lenalidomide	Multiple Myeloma	I	NCT01217203
Monalizumb (anti-NKG2A antibody)	In combination with durvalumab	Advanced solid tumors	I/II	NCT02671435
Cobolimab (TIM-3 Inhibitor)	In combination with dostarlimab	Resectable Stage III or Oligometastatic Stage IV Melanoma	II	NCT04139902
Domvanalimab (anti-TIGIT antibody)	Monotherapy or in combination with zimberelimab	Advanced solid tumors	I	NCT03628677
*TGF-β1 inhibitor therapy*				
Galunisertib	In combination with nivolumab	Advanced Refractory Solid Tumors	I/II	NCT02423343

Owing to the large number of trials in each category, example trials have been selected to illustrate the research and trials mentioned in this Review. Abbreviations: AML, acute myeloid leukemia; CIMI, cytokine-induced memory-like; HNSCC, head and neck squamous cell carcinoma; HSCT, hematopoietic stem cell transplantation; ICI, immune checkpoint inhibitor; NHL, non-Hodgkin lymphoma; NPC, nasopharyngeal carcinoma; NSCLC, non-small cell lung cancer; RCC, renal cell carcinoma; R/R, relapsed and/or refractory; UCB, umbilical cord blood.

## Data Availability

Data sharing not applicable.
